# Prevalence and profile of congenital heart disease and pulmonary hypertension in Down syndrome in a pediatric cardiology service

**DOI:** 10.1590/0103-0582201432218913

**Published:** 2014-06

**Authors:** Felipe Alves Mourato, Lúcia Roberta R. Villachan, Sandra da Silva Mattos

**Affiliations:** 1 Unidade de Cardiologia Materno-Fetal, Recife, PE, Brasil

**Keywords:** hypertension, pulmonary, heart defects, congenital, Down syndrome

## Abstract

**OBJECTIVE:**

To determine the frequence and profile of congenital heart defects in Down syndrome patients referred to a pediatric cardiologic center, considering the age of referral, gender, type of heart disease diagnosed by transthoracic echocardiography and its association with pulmonary hypertension at the initial diagnosis.

**METHODS:**

Cross-sectional study with retrospective data collection of 138 patients with Down syndrome from a total of 17,873 records. Descriptive analysis of the data was performed, using Epi-Info version 7.

**RESULTS:**

Among the 138 patients with Down syndrome, females prevailed (56.1%) and 112 (81.2%) were diagnosed with congenital heart disease. The most common lesion was *ostium secundum* atrial septal defect, present in 51.8%, followed by atrioventricular septal defect, in 46.4%. Ventricular septal defects were present in 27.7%, while tetralogy of Fallot represented 6.3% of the cases. Other cardiac malformations corresponded to 12.5%. Pulmonary hypertension was associated with 37.5% of the heart diseases. Only 35.5% of the patients were referred before six months of age.

**CONCLUSIONS:**

The low percentage of referral until six months of age highlights the need for a better tracking of patients with Down syndrome in the context of congenital heart disease, due to the high frequency and progression of pulmonary hypertension.

## Abstract (es)

**OBJETIVO:**

Determinar la frecuencia y el perfil de las cardiopatías congénitas en portadores de síndrome de Down atendidos en servicio de referencia de cardiología pediátrica, considerándose la edad del encaminamiento, el sexo, el tipo de cardiopatía diagnosticada al ecocardiografía transtorácica y su asociación con hipertensión pulmonar al diagnóstico inicial.

**MÉTODOS:**

Estudio de cohorte transversal con recolección retrospectiva de datos de 138 pacientes portadores de síndrome de Down de un total de 17.873 prontuarios. Los datos fueron sometidos al análisis descriptivo, utilizando el programa Epi-Info, versión 7.

**RESULTADOS:**

Entre los 138 pacientes con síndrome de Down, hubo mayor prevalencia del sexo femenino (56,1%) y 112 (81,2%) fueron diagnosticados con cardiopatía congénita. Entre las cardiopatías, la más común fue la comunicación interatrial *ostium secundum*, con frecuencia del 51,8%, seguida por el defecto del septo atrioventricular, con 46,4%. La comunicación interventricular estaba presente en el 27,7%, mientras que la tetralogía de Fallot representó solamente un 6,3% de los casos. Otras cardiopatías totalizaron 12,5%. La hipertensión pulmonar se asoció al 37,5% de las cardiopatías. Sólo un 35,5% de pacientes fueron encaminados al servicio hasta los seis meses de edad.

**CONCLUSIONES:**

La proporción de cardiopatías congénitas en portadores de síndrome de Down fue mayor en el servicio estudiado respecto a lo descripto en la literatura. Aún así, el bajo porcentaje de encaminamiento hasta los seis meses llama la atención para la necesidad de un mejor rastreo de los portadores de la síndrome en el contexto de las cardiopatías congénitas, teniendo en vista la alta frecuencia y la progresión de la hipertensión pulmonar.

## Introduction

Down Syndrome (DS) is characterized by a complete trisomy of chromosome 21 in 95% of cases, occurring in approximately one in every 700 live births^(^ 1 ^,^ 2 ^)^. This incidence may vary according to maternal age, affecting one in every 30 live births in mothers with age higher than 45 years^(^ 3 ^)^. The phenotype of DS includes muscular hypotonia, low height, dysmorphic facial features, heart malformations, and cognitive deficits^(^ 4 ^)^, with variable characteristics among carriers.

Congenital heart diseases occurr in 40 to 60% of individuals with Down syndrome, being one of the primary causes of morbimortality^(^ 5 ^)^, especially in the first 2 years^(^ 3 ^,^ 6 ^)^. On the other hand, symptoms or signs of these heart diseases may be absent in the fisrt days, what leads to a late diagnosis. This may be determining in the development of heart failure, penumonia, cardiac arrhythmias, or pulmonary hypertension.

Pulmonary hypertension (PH) is characterized by a continuous increase in vascular pressure that progressively leads to a remodeling of the pulmonary vessels and to right ventricular failure^(^ 7 ^)^. Heart diseases that have left-right shunt with increased pulmonary blood flow (such as interatrial and interventricular communication) lead more easily to situations of PH. Its symptoms are usually nonspecific (progressive dyspnea on exertion, angina chest pain, among others). Thus, the early diagnosis of congenital heart disease is essential to prevent or treat PH in the early stages. In DS, the investigation of such diseases is mandatory because of the high incidence of cardiac malformations and the rapid progress of PH^(^ 8 ^)^ in this population. 

Therefore, considering the importance of congenital heart disease for children with DS, this study sought to determine the prevalence and the profile of these diseases in these patients. We also verified the presence and severity of PH at diagnosis in a referral center for pediatric cardiology in the state of Pernambuco.

## Method

We conducted a cross-sectional, descriptive and retrospective study of patients with DS treated in a pediatric cardiology referral center between 2005 and 2010. Patients were referred by pediatricians in the municipality of Recife and surroundings, without a predetermined flow of referral of patients with chromosomal disorders. It is worth mentioning that, during the study period, 1,236 patients with DS were born in the state of Pernambuco. 

Data were collected from the database of the service and sorted according to the confirmation of the syndrome (evident phenotype or karyotype). Among a total of 17,873 patients treated at the referral center, 138 had DS and were selected for the study. 

The respective medical charts were reviewed by collecting the following data: presence or absence of congenital heart disease (diagnosed by transthoracic echocardiography), type of disease, gender, age at referral, presence or absence of pulmonary hypertension at the first echocardiography performed in the service (pulmonary systolic pressure above 25mmHg at rest on echocardiography), and reason for referral. According to the referral reasons, patients were allocated into two groups: with and without suspicion of congenital heart disease. The records obtained were submitted to analysis of frequencies using the Epi-Info program, version 7. To differentiate the *ostium secundum* atrial septal defect from a patent foramen ovale, we used the documentation of a drop out in the atrial septum in the subcostal view (where the defect shows well-defined edges and no echoes in the fossa oval area) associated to the visualization of the interatrial shunt by Doppler and presence of signs of volume overload.

Subsequently, those who presented PH had their echocardiographic reports analyzed for determination of pulmonary artery pressure (PAP). This analysis referred to the first echocardiogram at the service, always performed by pediatric cardiologists. Patients whose echocardiogram was performed outside the service were excluded from the analysis. Then, the degree of PH was classified as mild (PAP between 25 and 40mmHg), moderate (PAP between 41 and 55mmHg) or severe (PAP higher than 55mmHg), based on the echocardiogram. The diagnosis of PH was established by estimating the peak systolic gradient of the shunt between the ventricles, through the Bernoulli equation, in patients with intracardiac defects (atrial, ventricular or atrioventricular septal defects). In cases with patent ductus arteriosus, we used the shunt between the aorta and the pulmonary artery. The analysis of the regurgitation jet of the right atrioventricular valve was used only in cases where there was no possibility of shunt between the left ventricle and the right atrium. 

## Results

Among the 138 analyzed patients, 112 presented congenital heart disease (81.2%), who were predominantly female (56.1%). Only 23.2% were referred without congenital signs of heart disease, with 43.8% subsequently diagnosed with heart disease. 

Table 1 shows the prevalence of different types of congenital heart disease in patients with DS. The most common was *ostium secundum atrial septal defect* (ASD) with 51.78%, followed by atrioventricular septal defect (AVSD), with 45.5% - with its complete form representing 22.3% -, and patent ductus arteriosus (PDA), with 34.8%. Tetralogy of Fallot represented only 6.3% of cases. It is important to highlight that many patients presented more than one structural heart defect concomitantly.

**Table 1:**
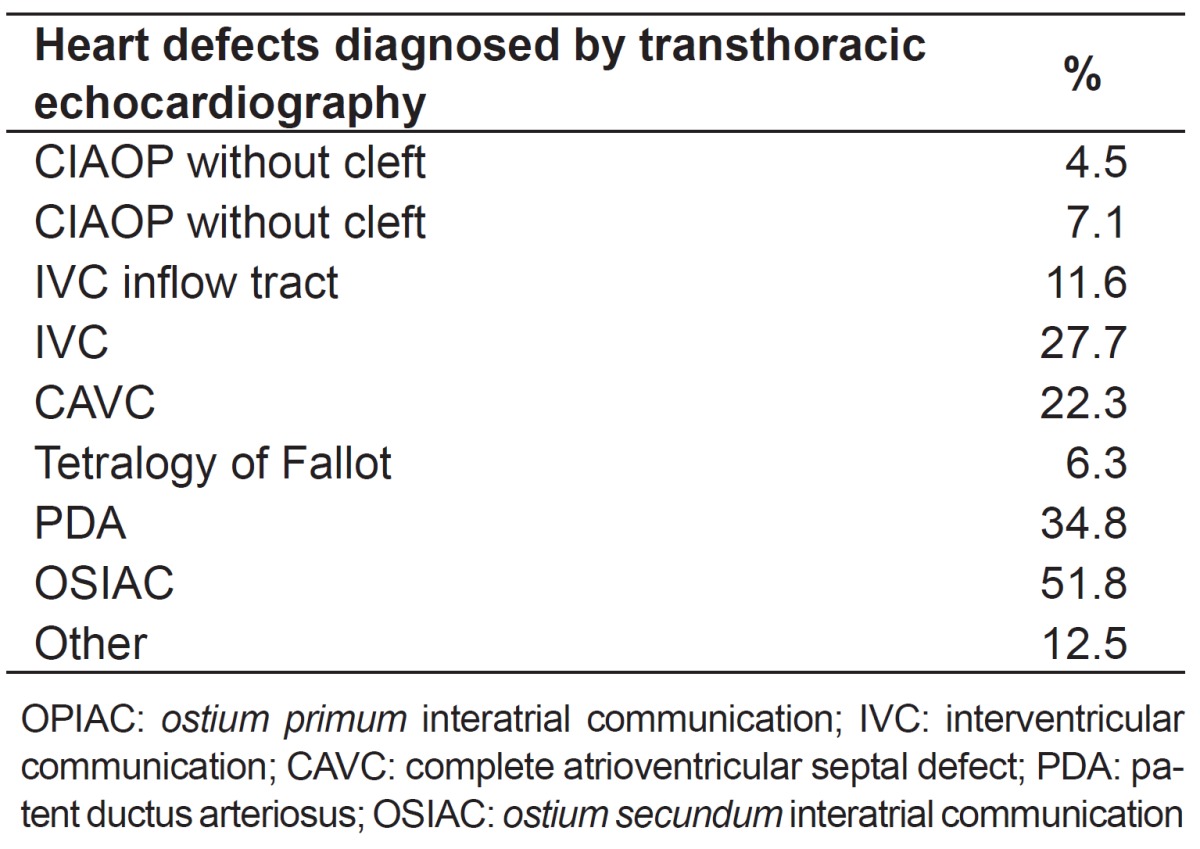
Prevalence of congenital heart disease in children with Down syndrome

As to the age at referral, patients were divided into before and after 6 months of age. Only a minority (35.5%) was referred before 6 months. The most common cardiovascular defects in this group were ASD (59.2%), PDA (40.8%) and partial atrioventricular septal defect (PAVSD - 30.6%).

Pulmonary hypertension was found in 42 (37.5%) patients diagnosed with congenital heart disease. Among these, around 1/3 of diagnostic echocardiograms were not performed in the service, so they were excluded from the analysis of the degree of pulmonary hypertension at diagnosis. In the remaining 2/3, we obtained: 21.4% of mild PH; 21.4% of moderate PH and 57.1% of severe PH. Among patients with PH, 11 were referred before 6 months of age, two with mild pulmonary arterial hypertension (PAH), 2 with moderate PAH, and 7 with severe PAH. In the group with referral after 6 months, four presented mild PAH, four moderate PAH and nine presented severe PAH. 

In cases with mild PH, the most frequent diseases were the AVSD and ASD, affecting from six to five cases, respectively. All patients with moderate PAH had AVSD, associated to PDA is three cases. In patients with severe PAH, AVSD was the most frequent (10 cases), followed by VSD (eight cases). More details are found in Table 2.

**Table 2:**
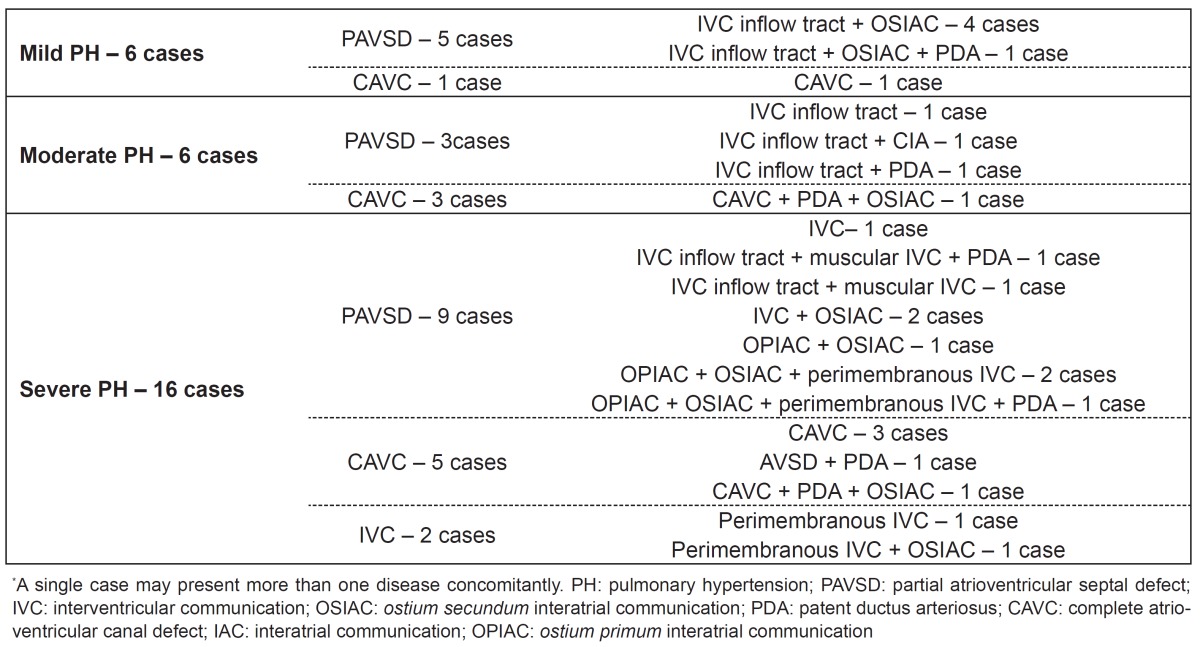
Relationship between the degree of pulmonary hypertension, number of cases, and cardiac lesions

## Discussion

Among the patients with DS referred to the center, 81.2% showed changes at the echocardiogram, which was a higher frequency than that reported in the literature, between 40 and 60%^(^ 2 ^,^ 5 ^,^ 9 ^)^. This finding is justified by the fact that the study site is a reference center, receiving patients who were already screened. Of those who were referred only due to DS (that is, without clinical suspicion of congenital heart disease), 43.8% suffered from some heart disease. This demonstrates that, often, the disease does not show clear signs and symptoms, especially in the first days of life.

The higher prevalence of AVSD, ASD, VSD, and PDA in individuals with DS is widely reported in the literature. However, studies differ as to the frequency of each, being AVSD^(6) ^more prevalent in some researches, while, in others, VSD is the most common^(^ 10 ^)^. In this study, the ASD showed the highest prevalence (51.8%). This difference may be due to the fact that this study used outpatients, that is, those in better clinical conditions. Therefore, we may have underestimated the amount of serious defects, such as the AVSD, in comparison to studies covering the entire population with DS. Nevertheless, the most common diseases found are consistent with the same group of diseases reported in the literature.

Only 35.5% of patients with DS were referred before six months of age for investigation of possible congenital heart disease. This is worrying, since early diagnosis coupled with effective surgical treatment is mainly responsible for the decrease in morbidity and mortality in this population^(^ 11 ^-^ 13 ^)^. 

In patients with pulmonary hypertension, the severe form was present in 57.1%, while the milder forms were equally divided among the rest. The majority of cases is explained by the fact that the disease leads to an increased pulmonary blood flow and, consequently, to PH. Moreover, it has been reported that patients with DS demonstrate early progression to PH when they present with left-to-right shunt lesions^(^ 14 ^)^. However, it should be highlighted that individuals with DS may have PH for various causes such as chronic airway obstruction, abnormal growth of the pulmonary vasculature, alveolar hypoventilation, decreased number of alveoli, thinner pulmonary arterioles, among others^(^ 14 ^)^. 

However, only a minority of patients was referred to the service before 6 months of age, preventing early diagnosis. This fact may hinder the possibility of heart surgery because PH can evolve to a scenario in which surgery is contraindicated (PH by hyperesistance), further increasing mortality in these patients. Furthermore, most patients had severe PH, emphasizing even more the importance of the early diagnosis. This fact is described in some studies that justify the finding by the possibility of endothelial dysfunction in such patients^(^ 15 ^)^. Moreover, the incidence of PAH in neonates with DS is much larger (up to 50 times more than those without the syndrome)^(^ 16 ^)^, which makes them more prone to progress to Eisenmenger syndrome than other groups^(^ 17 ^)^.

It is noteworthy that this study has limitations. The fact that this was a retrospective study decreases quality in obtaining the necessary information, which was minimized by the adoption of electronic medical records and the fact that only the echocardiograms performed by pediatric cardiologists within the service were analyzed. However, the use of the non-invasive assessment, rather than cardiac catheterization to establish the diagnosis of PH was also a limitation of the study. On the other hand, the study is valid due to its considerable sample size and for the relative small number of studies associated to DS and PAH in Brazil. 

Therefore, the prevalence of congenital heart defects in individuals with DS was higher in the health service studied in comparison to other studies, which can be explained by the fact that the service is a referral center. Still, the low percentage of referrals before the age of 6 months reinforces the need for better tracking of patients with DS. This approach becomes imperative when considering the high frequency and the evolution to PH in these patients.
