# Supplementary material for: Prevalence and profile of congenital heart disease and pulmonary hypertension in Down syndrome in a pediatric cardiology service
Source: Rev Paul Pediatr. 2014 Jun;32(2):159–63. doi: 10.1590/0103-0582201432218913 (PMC4183023; doi:10.1590/0103-0582201432218913)
Supplement: Translation

# Translation: Prevalência e perfil das cardiopatias congênitas e hipertensão pulmonar na síndrome de Down em serviço de cardiologia pediátrica

**Authors:** Felipe Alves Mourato, Lúcia Roberta R. Villachan, Sandra da Silva Mattos

**Affiliations:** 1 Unidade de Cardiologia Materno-Fetal, Recife, PE, Brasil

**Keywords:** hipertensão pulmonar, cardiopatias congênitas, síndrome de Down

## Abstract

**OBJETIVO:**

Determinar a frequência e o perfil das cardiopatias congênitas em portadores de síndrome de Down atendidos em serviço de referência de cardiologia pediátrica, considerando-se a idade do encaminhamento, o sexo, o tipo de cardiopatia diagnosticada ao ecocardiograma transtorácico e sua associação com hipertensão pulmonar ao diagnóstico inicial.

**MÉTODOS:**

Estudo de corte transversal com coleta retrospectiva de dados de 138 pacientes portadores de síndrome de Down de um total de 17.873 prontuários. Os dados foram submetidos à análise descritiva, utilizando-se o programa Epi-Info, versão 7.

**RESULTADOS:**

Entre os 138 pacientes com síndrome de Down, houve maior prevalência do sexo feminino (56,1%) e 112 (81,2%) foram diagnosticados com cardiopatia congênita. Entre as cardiopatias, a mais comum foi a comunicação interatrial *ostium secundum*, com frequência de 51,8%, seguida pelo defeito do septo atrioventricular, com 46,4%. A comunicação interventricular estava presente em 27,7%, enquanto a tetralogia de Fallot representou 6,3% dos casos. Outras cardiopatias totalizaram 12,5%. A hipertensão pulmonar associou-se a 37,5% das cardiopatias. Apenas 35,5% dos pacientes foram encaminhados ao serviço até os seis meses de idade.

**CONCLUSÕES:**

O baixo percentual de encaminhamento até os seis meses chama atenção para a necessidade de um melhor rastreamento dos portadores da síndrome no contexto das cardiopatias congênitas, tendo em vista a alta frequência e a progressão da hipertensão pulmonar.

## Introdução

A Síndrome de Down (SD) caracteriza-se pela trissomia completa do cromossomo 21 em 95% dos casos, ocorrendo aproximadamente em um a cada 700 nascidos vivos^(^ ^1^ ^,^ ^2^ ^)^. Essa incidência pode variar de acordo com a idade materna, atingindo um a cada 30 nascidos vivos em mães com idade superior a 45 anos^(^ ^3^ ^)^. O fenótipo da SD inclui hipotonia muscular, baixa estatura, dismorfismos faciais, malformações cardíacas e déficits cognitivos^(^ ^4^ ^)^, sendo essas características variáveis entre os portadores.

As cardiopatias congênitas ocorrem em 40 a 60% dos portadores da síndrome, destacando-se na sua morbimortalidade^(^ ^5^ ^)^, principalmente nos dois primeiros anos^(^ ^3^ ^,^ ^6^ ^)^. Por outro lado, sintomas ou sinais dessas cardiopatias podem estar ausentes nos primeiros dias de vida, o que acaba por levar ao diagnóstico tardio. Isso pode ser determinante no desenvolvimento de insuficiência cardíaca congestiva, pneumonia, arritmias cardíacas ou hipertensão pulmonar.

A hipertensão pulmonar (HP) caracteriza-se pelo aumento contínuo da pressão vascular que, progressivamente, leva a uma remodelação dos vasos pulmonares e à falência ventricular direita^(^ ^7^ ^)^. As cardiopatias que apresentam *shunt* esquerda-direita com hiperfluxo pulmonar (como comunicação interatrial e interventricular) levam mais facilmente a quadros de HP. Seus sintomas geralmente são inespecíficos (dispneia progressiva aos esforços, dor torácica anginosa, entre outros). Dessa forma, o diagnóstico precoce de cardiopatias congênitas é essencial para evitar ou tratar a HP em fases mais precoces. Na SD, a investigação de tais entidades é mandatória devido à alta incidência de malformações cardíacas e à evolução mais rápida da HP^(^ ^8^ ^)^ nessa população. 

Logo, considerando-se a importância das cardiopatias congênitas para a criança com SD, este trabalho buscou determinar a prevalência e perfil dessas cardiopatias nesses pacientes. Verificou também a presença e a gravidade da HP no momento do diagnóstico em um serviço de referência em cardiologia pediátrica no estado de Pernambuco.

## Método

Realizou-se um estudo de corte transversal, descritivo e retrospectivo, de pacientes com SD atendidos em serviço de referência em cardiologia pediátrica entre 2005 e 2010. Os pacientes foram encaminhados por pediatras na cidade do Recife e cidades circunvizinhas, sem haver um fluxo pré-estabelecido de encaminhamento de pacientes com cromossomopatia. Vale lembrar que, no período do estudo, nasceram 1.236 crianças com SD no estado de Pernambuco. 

Os dados foram colhidos do banco de dados do serviço e triados de acordo com a confirmação da síndrome (fenótipo evidente ou cariótipo). De um total de 17.873 pacientes atendidos no serviço de referência, 138 possuíam SD e foram selecionados para o estudo. 

Os respectivos prontuários foram revisados, coletando-se os seguintes dados: presença ou não de cardiopatia congênita (diagnosticada por meio de ecocardiograma transtorácico), tipo da cardiopatia, sexo, idade do encaminhamento ao serviço, presença ou não de hipertensão pulmonar ao primeiro ecocardiograma realizado no serviço (pressão sistólica pulmonar acima de 25mmHg em repouso no ecocardiograma) e motivo do encaminhamento. De acordo com os motivos de encaminhamento, alocaram-se os pacientes em dois grupos: com e sem suspeita clínica de cardiopatia congênita. Para diferenciar a comunicação interatrial *ostium secundum* de um foramen oval patente, utilizou-se a documentação de solução de continuidade do septo interatrial no corte subcostal (onde o defeito se apresenta com bordas bem definidas e ausência de ecos na membrana da fossa oval), associada à visualização do *shunt* interatrial pelo Doppler e presença de repercussões hemodinâmicas do defeito.

Logo depois, aqueles que apresentavam HP tiveram seus laudos ecocardiográficos analisados para determinação da pressão na artéria pulmonar (PAP). Essa análise referiu-se ao primeiro ecocardiograma do paciente realizado no serviço, sempre por cardiologistas pediátricos. Excluíram-se da análise os pacientes cujo ecocardiograma diagnóstico foi realizado fora do serviço. Em seguida, o grau da HP foi classificado em leve (PAP entre 25 e 40mmHg), moderada (PAP entre 41 e 55mmHg) ou grave (PAP maior do que 55mmHg), com base no ecocardiograma. Estabeleceu-se o diagnóstico de HP por meio da estimativa dos gradientes de pico sistólico do *shunt *entre os ventrículos, pela equação de Bernoulli, nos pacientes com defeitos intracardíacos (comunicação interatrial, comunicação interventricular - CIV - ou defeito do septo atrioventricular). Nos casos com persistência do canal arterial, utilizou-se o *shunt *entre a artéria aorta e a pulmonar. A análise do jato da regurgitação da valva atrioventricular direita foi utilizada somente nos casos em que não existia possibilidade de *shunt *entre o ventrículo esquerdo e o átrio direito.

A análise estatística foi descritiva por meio do software Epi-Info, versão 7.

## Resultados

Dos 138 pacientes analisados, 112 apresentaram cardiopatia congênita (81,2%), com predomínio do sexo feminino (56,1%). Apenas 23,2% foram encaminhados sem suspeita clínica de cardiopatia, sendo 43,8% posteriormente diagnosticados como portadores de cardiopatia congênita. 

A Tabela 1 demonstra a prevalência dos tipos de cardiopatia congênita nos portadores de SD. A mais comum foi a comunicação interatrial *ostium secundum* (CIAOS) com 51,8%, seguida pelo defeito do septo atrioventricular (DSAV), com 45,5% - com sua forma total representando 22,3% -, e pela persistência do canal arterial (PCA), com 34,8%. A tetralogia de Fallot representou 6,3% dos casos. É importante salientar que boa parte dos pacientes apresentou mais de um defeito estrutural cardíaco concomitantemente.

**Tabela 1:** Cardiopatias congênitas diagnosticadas ao ecocardiograma transtorácico nas crianças com síndrome de Down

Quanto à idade do encaminhamento, os pacientes foram divididos em antes e após os seis meses de idade. Uma minoria (35,5%) foi encaminhada antes dos seis meses. Os defeitos cardiovasculares mais frequentes nesse grupo foram a CIAOS (59,2%), a PCA (40,8%) e o defeito do septo atrioventricular parcial (DSAVP - 30,6%).

A hipertensão pulmonar foi encontrada em 42 (37,5%) pacientes diagnosticados com cardiopatia congênita. Nestes, cerca de 1/3 dos ecocardiogramas diagnósticos não foram realizados no serviço, tendo sido excluídos da análise do grau de hipertensão pulmonar ao diagnóstico. Nos 2/3 restantes, obteve-se: 21,4% de HP leve; 21,4% de HP moderada e 57,1% de HP grave. Dos pacientes com HP, 11 foram ecaminhados antes dos seis meses de idade, sendo dois com hipertensão arterial pulmonar (HAP) leve, dois com HAP moderada e sete com HAP grave. No grupo com encaminhamento acima dos seis meses, quatro apresentaram HAP leve, quatro HAP moderada e nove apresentaram HAP grave. 

Nos casos com HP leve, as cardiopatias mais encontradas foram o DSAV e a CIAOS, acometendo seis e cinco casos, respectivamente. Nos casos de HAP moderada, o DSAV foi encontrado em todos, associado à PCA em três deles. Na HAP grave, o DSAV foi o mais frequente (dez casos), seguido pela CIV (oito casos). Mais detalhes encontram-se no Quadro 1.

**Quadro 1:** Relação entre grau de hipertensão pulmonar, quantidade de casos e cardiopatias presentes

## Discussão

Dos pacientes com SD encaminhados ao centro de referência, 81,2% mostraram alterações ao ecocardiograma, sendo tal frequência superior à relatada na literatura, entre 40 e 60%^(^ ^2^ ^,^ ^5^ ^,^ ^9^ ^)^. Justifica-se tal achado pelo fato de o local do estudo ser um centro de referência, recebendo pacientes já triados. Dos que foram encaminhados unicamente pela SD (ou seja, sem suspeita clínica de cardiopatia congênita), 43,8% eram portadores de alguma cardiopatia. Isso demonstra que, muitas vezes, a cardiopatia não apresenta sinais e sintomas evidentes, principalmente nos primeiros dias de vida.

A maior prevalência de DSAV, CIAOS, CIV e PCA em portadores de SD é amplamente relatada na literatura. Entretanto, os estudos divergem quanto às frequências de cada um, sendo o DSAV^(6) ^mais prevalente em algumas pesquisas, enquanto, em outras, a CIV é a que mais ocorre^(^ ^10^ ^)^. Neste trabalho, a CIAOS foi a que apresentou maior prevalência (51,8%). Tal diferença pode se dever ao fato de este estudo contar com pacientes ambulatoriais, ou seja, aqueles em melhores condições clínicas. Dessa forma, é possível que tenha ocorrido uma subestimação da quantidade de cardiopatias mais graves, como o DSAV, em comparação com estudos que abrangeram toda a população com SD. Apesar disso, as cardiopatias mais frequentes encontradas condizem com o mesmo grupo relatado na literatura.

Somente 35,5% dos pacientes com SD foram encaminhados antes dos seis meses de idade para investigação de possível cardiopatia congênita. Isso é preocupante, já que o diagnóstico precoce aliado ao tratamento cirúrgico efetivo é o principal responsável pela diminuição da morbimortalidade nessa população^(^ ^11^ ^-^ ^13^ ^)^. 

Nos pacientes com HP, a forma grave estava presente em 57,1%, enquanto as formas mais leves apresentaram-se igualmente divididas entre os demais. A maioria dos casos é explicada pela cardiopatia levar a um hiperfluxo pulmonar e, consequentemente, à HP. Ademais, já se descreveu que pacientes com SD demonstram evolução precoce para HP quando apresentam cardiopatia com hiperfluxo pulmonar^(^ ^14^ ^)^. Todavia, vale salientar que os portadores de SD podem apresentar HP por diversas causas, como obstrução crônica da via aérea, crescimento anormal da vascularização pulmonar, hipoventilação alveolar, número diminuído de alvéolos, arteríolas pulmonares mais finas, entre outras^(^ ^14^ ^)^. 

Entretanto, somente a minoria dos pacientes foi encaminhada ao serviço antes dos seis meses de idade, impossibilitando o diagnóstico precoce. Esse fato pode inviabilizar a cirurgia cardíaca, pois a HP pode evoluir para um quadro no qual a intervenção cirúrgica é contraindicada (HP por hiperresistência), elevando ainda mais a mortalidade nesses pacientes. Além disso, a maior parcela dos pacientes tinha HP grave, aumentando ainda mais a importância do diagnóstico precoce. Esse achado é descrito em alguns estudos que discutem a possibilidade de disfunção endotelial em tais pacientes^(^ ^15^ ^)^. Além disso, a incidência de HAP em neonatos com SD é bem maior (até 50 vezes mais do que aqueles sem a síndrome)^(^ ^16^ ^)^, sendo mais comum a evolução para síndrome de Eisenmenger do que em outros grupos^(^ ^17^ ^)^.

Vale ressaltar que o estudo possui limitações. O fato de ser retrospectivo diminui a qualidade da obtenção das informações necessárias, o que foi minimizado com a adoção do prontuário eletrônico e pelo fato de terem sido analisados apenas os ecocardiogramas realizados por cardiologistas pediátricos do serviço, embora a utilização do exame, em vez do cateterismo, para diagnóstico da HP seja também uma limitação do trabalho. Por outro lado, o estudo torna-se válido pela sua população amostral considerável e pela pouca quantidade de trabalhos que associam SD e HAP em nosso meio. 

Logo, a prevalência de cardiopatias congênitas em portadores de SD foi maior no serviço estudado em comparação a outros trabalhos, o que pode ser explicado pelo serviço ser de referência. Ainda assim, o baixo percentual de encaminhamento até os seis meses reforça a necessidade de um melhor rastreamento dos portadores de SD. Essa conduta torna-se imperativa quando se considera a alta frequência e evolução para HP nesses pacientes.
